# Circulating adipokines and insulin resistance in subjects with combined cardiac and metabolic syndrome X

**DOI:** 10.1186/s13098-015-0078-4

**Published:** 2015-09-24

**Authors:** Kae-Woei Liang, Wen-Jane Lee, Wen-Lieng Lee, Ying-Chieh Liao, Kuo-Yang Wang, I.-Te Lee, Jun-Sing Wang, Wayne H.-H. Sheu

**Affiliations:** Cardiovascular Center, Taichung Veterans General Hospital, 1650 Taiwan Boulevard, Sec. 4, Taichung, 40705 Taiwan; School of Medicine, National Yang Ming University, Taipei, Taiwan; Department of Medicine, China Medical University, Taichung, Taiwan; Department of Medical Research, Taichung Veterans General Hospital, Taichung, Taiwan; Tung-Hai University, Taichung, Taiwan; Taichung Tzu Chi Hospital, Taichung, Taiwan; Department of Medicine, Tzu Chi University School of Medicine, Hualian, Taiwan; Department of Medicine, Chung Shan Medical University, Taichung, Taiwan; Division of Endocrinology and Metabolism, Department of Medicine, Taichung Veterans General Hospital, 1650 Taiwan Boulevard, Sec. 4, Taichung, 40705 Taiwan; Institute of Biomedical Sciences, National Chung Hsing University, Taichung, Taiwan; School of Medicine, National Defense Medical Center, Taipei, Taiwan

**Keywords:** Adiponectin, Cardiac syndrome X (CSX), Combined double syndrome X, Leptin, Metabolic syndrome X (MetX)

## Abstract

**Background:**

Cardiac syndrome X (CSX) is characterized by angina pectoris but with patent coronary arteries. Our previous study showed that CSX subjects had decreased serum adiponectin but higher leptin and insulin resistance (IR). However, few studies have investigated circulating adipokines and IR in subjects with combined metabolic syndrome X (MetX) and CSX.

**Methods:**

Fifty-nine subjects with CSX were retrospectively enrolled from our cardiac catheterization patient databank. Fifty-four subjects with valvular heart disease or arrhythmia and with normal coronary angiograms were recruited as the non-CSX comparison group. The study subjects were reclassified according to the presence or absence of MetX. Circulating adipokines and degree of IR were measured.

**Results:**

Subjects with combined MetX and CSX had a significantly higher HOMA-IR, a higher circulating leptin level (median 8.7 vs. 3.3 ng/mL, p < 0.001), but a lower circulating adiponectin level (median 2.8 vs. 12.3 μg/mL, p < 0.001) than those without MetX and CSX. In pairwise comparisons, combined MetX and CSX subjects had a similar circulating adipokines and IR index as those who had only either one syndrome X. In a multivariate regression analysis, serum triglycerides (odds ratio 1.011, p = 0.024) and hypertension (odds ratio 14.453, p = 0.003) were independently associated with diagnosis of combined MetX and CSX.

**Conclusions:**

Combined MetX and CSX had a significantly higher HOMA-IR, a higher circulating leptin but a lower circulating adiponectin level than those without MetX and CSX. Combined syndrome X did not confer more changes on adipokines or IR index comparing with those with only one syndrome X.

## Background

Metabolic syndrome X (MetX) is a special cluster of atherosclerotic risk factors associated with obesity and insulin resistance (IR) [1]. Subjects with MetX have a higher incidence of cardiovascular diseases and increased cardiovascular mortality [2–5]. We and others have demonstrated that patients of MetX had decreased circulating adiponectin but increased leptin and a higher inflammatory status [6–8].

Subjects with cardiac syndrome X (CSX) have angina like symptoms with evidence of ischemia in stress electrocardiogram or isotope perfusion scan but with patent epicardial coronary arteries on coronary angiogram [9–11]. The proposed mechanisms underlying CSX included endothelial dysfunction with impaired vaso-dilatory reserve in micro-vascular beds, inflammation, IR, estrogen deficiency, or oxidative stress [12, 13].

The two syndrome Xs, CSX and MetX, shared some common features and pathogenesis. In one study, hyper-insulinemia during oral glucose tolerance test was more prominent in the CSX group than in controls, which implies that IR might contribute to the micro-vascular angina [14]. In addition, Jadhav et al. [15] reported that women with CSX more commonly had MetX and related adiposity, metabolic and inflammatory derangements. Using hyper-insulinemia and the euglycemia clamp test, other investigators found that subjects with CSX or MetX had higher degree of IR as compared to controls [16].

Several clinical studies have found decreased circulating adiponectin level in populations with hypertension, obesity, MetX, type 2 DM, and coronary artery disease (CAD) [17–19]. Increased food intake and IR have been shown to increase plasma leptin levels and lead to leptin resistance in tissue, which is very common in obesity and MetX [20]. Elevated serum leptin level is also an independent risk factor for development of atherosclerotic cardiovascular disease and MetX [21]. Our previous study showed that CSX subjects had lower circulating adiponectin but higher leptin and higher leptin/adiponectin ratio than those of the control group [22]. There was also a report showing that women with CSX had a higher circulating leptin than the healthy control group after body mass index correction [15].

To date, few studies have investigated circulating adipokines and IR in subjects with double syndrome X that is, combined MetX and CSX, or either syndrome X alone. Moreover, the usefulness of the clinical variables associated with the diagnosis of combined syndrome X among subjects who have undergone coronary angiogram but without stenosis remained unexplored. The aims of the present study were to compare circulating adipokine levels in subjects with either MetX or CSX or both and determine what component of MetX or other atherosclerotic or cytokine markers are associated with the presence of combined MetX and CSX.

## Methods

### Study population

**Fig. 1:**
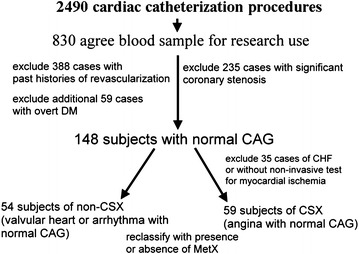
Flow chart of the study enrollment protocol. *CAG* coronary angiogram, *CHF* congestive heart failure, *DM* diabetes mellitus, *CSX* cardiac syndrome X, *MetX* metabolic syndrome X

From January 2010 to February 2011, a total of 2490 cardiac catheterization procedures, including coronary angiograms, percutaneous coronary or peripheral vascular interventions, electrophysiological studies, catheter radiofrequency ablations and pacemaker implantations, were performed at our catheterization laboratories. Among the patients who underwent these procedures, 830 patients agreed to donate a blood sample for research purposes and signed an informed consent form. Subjects with significant coronary stenosis (N = 235) (Gensini score >0 or Syntax score >0) [23, 24] or past histories of surgical or percutaneous coronary revascularization before the index admission (N = 388) were excluded. After excluding 59 additional subjects with overt DM requiring medical control, the remaining 148 non-diabetic subjects with normal coronary angiograms were enrolled for analysis. Among these 148 subjects, 35 patients were excluded because of decompensated congestive heart failure or without non-invasive tests for myocardial ischemia (Fig. 1). Fifty-nine patients who had chest pain with clinical suspicion of angina pectoris and had undergone non-invasive tests suggesting myocardial ischemia were defined as having CSX, while 54 patients with valvular heart disease or arrhythmia who underwent coronary angiograms for pre-operative or peri-procedural study without mention of chest pain were classified as the non-CSX group for comparisons (Fig. 1) [22]. Some of the study results have been published previously [22]. The 59 CSX and 54 non-CSX subjects were subsequently regrouped according to the presence or absence of MetX (Fig. 1). The definition of MetX was made according to the ATP III criteria [25], with modification of the central obesity criterion into body mass index (BMI) equal to or greater than 27 kg/m^2^ [26]. Those who did not have MetX or CSX served as the control group. We retrospectively reviewed all patients’ angiographic images, catheterization reports, and medical chart records. The study protocol was approved by the Human Research Review Committee of Taichung Veterans General Hospital (Taichung, Taiwan).

### High-sensitivity C-reactive protein (hs-CRP), fasting blood glucose, insulin, and the definition of HOMA insulin-resistance index

Serum high-sensitivity C-reactive protein (hs-CRP) was determined by particle-enhanced immunoturbidimetry (Good Biotech Corp., Taichung, Taiwan) [8]. The intra- and inter-assay coefficients of variance were 0.75 and 1.89 %, respectively. The calculated low-density lipoprotein (LDL) cholesterol concentration was estimated by the formula devised by Friedewald et al. [27]. Serum insulin was determined using a commercially available assay kit (IMMULITE, I-2000, EURO/Diagnostic Products Corporation, Gwynedd, UK) [28]. The inter- and intra-assay coefficients of variation for insulin (range 10.7–439 μU/mL) were 4.3 and 5.4 %, respectively. Homeostasis model assessment (HOMA) index was calculated (fasting glucose mg/dL × fasting insulin μU/mL/405) as an index of IR.

### Measurements of circulating serum adipokines

Serum adiponectin and leptin levels were determined by the enzyme-linked immunosorbent assay (ELISA) kits (R & D Systems Inc. Minneapolis, MN, USA). The intra- and inter-assay coefficients of variation for adiponectin were 3.53 and 6.50 %, respectively, with a minimum detectable concentration of 0.079–0.891 ng/mL. The intra- and inter-assay coefficients of variation for leptin were 3.17 and 4.37 %, respectively, with a minimum detectable concentration of lower than 7.8 pg/mL.

### Statistical analysis

Continuous variables are expressed as median (interquartile range, 25th percentile to 75th percentile) because of a non-normal distribution and categorical data are expressed as percentages. Differences in continuous variables among the CSX, MetX, combined CSX and MetX and control groups were assessed by the Kruskal–Wallis test. The post hoc pairwise comparisons were analyzed by the Mann–Whitney U test (with a Bonferroni correction, p cut-off value for significance <0.0083). Categorical variables were compared by Chi square test or Fisher’s exact test as indicated. Binary logistic regression analyses were used to test significant variables associated with the diagnosis of “combined CSX and MetX”, “CSX without MetX” or “MetX without CSX”. The SPSS (version, 12.1) statistical software package (SPSS, Inc., Chicago, IL, USA) was used for all calculations. A two-tailed p value of less than 0.05 was considered statistically significant.

## Results

### Baseline demographic data, inflammatory marker and lipid profiles in subjects with and without cardiac or metabolic syndrome X

The gender and age were similar among the four groups (Table 1). Subjects with combined syndrome X had a greater BMI than the control (p < 0.001). The inflammatory marker, hs-CRP, was similar among the four groups (Table 1). Total cholesterol and triglycerides were significantly different among the four groups and were significantly higher in subjects with combined MetX and CSX (p < 0.001 vs. without MetX and CSX group) (Table 1).

**Table 1:** Baseline demographic data of subjects with and without cardiac or metabolic syndrome X

	CSX (−) MetX (−) (N = 39)	CSX (+) MetX (−) (N = 35)	CSX (−) MetX (+) (N = 15)	CSX (+) MetX (+) (N = 24)	*p* ^a^	*p* ^b^
Gender (M/F)	25/14	25/10	8/7	14/10	0.593	
Age (years)	59 (49, 68)	56 (50, 65)	54 (46, 61)	55 (50, 66)	0.638	0.655
Hypertension (N) (%)	8 (20.5 %)	15 (42.9 %)	9 (60 %)	22 (91.7 %)	<0.001	
Body mass index (kg/m^2^)	23.8 (22.4, 26.0)	25.2 (23.9, 27.6)	28.6 (25.5, 31.5)	27.7 (24.6, 30.8)	<0.001	<0.001
Current smoking (N) (%)	2 (5.1 %)	6 (17.1 %)	4 (26.7 %)	9 (37.5 %)	0.012	
Total cholesterol (mg/dL)	151 (134, 170)	164 (146, 193)	166 (135, 196)	185 (159, 211)	0.004	<0.001
Calculated LDL-C (mg/dL)	83 (74, 104)	96 (83, 120)	93 (67, 106)	97 (76, 134)	0.105	0.057
HDL-C (mg/dL)	48 (41, 60)	51 (46, 56)	42 (36, 44)	43 (38, 48)	<0.001	0.015
Triglycerides (mg/dL)	86 (51, 115)	83 (64, 113)	156 (116, 192)	203 (145, 240)	<0.001	<0.001
Fasting plasma glucose (mg/dL)	94 (88, 112)	95 (89, 113)	110 (102, 126)	106 (93, 128)	0.012	0.015
Fasting serum insulin (µIU/mL)	4.5 (2.9, 9.9)	8.4 (4.9, 10.6)	9.2 (5.9, 16.7)	9.4 (5.1, 19.9)	0.022	0.012
Hs-CRP (mg/dL)	0.127 (0.057, 0.344)	0.079 (0.036, 0.297)	0.220 (0.152, 0.530)	0.138 (0.082, 0.318)	0.180	0.661
Medication use						
Use of statin (N) (%)	3 (7.7 %)	2 (5.7 %)	0	3 (12.5 %)	0.506	
Use of ACEI or ARB (N) (%)	12 (30.8 %)	7 (20 %)	3 (20 %)	10 (41.7 %)	0.270	

Data are expressed as median (interquartile range, 25th percentile to 75th percentile) because of a non-normal distribution

*ACEI* angiotensin converting enzyme inhibitors, *ARB* angiotensin receptor blockers, *CSX* cardiac syndrome X, *HDL-C* high-density lipoprotein cholesterol, *Hs-CRP* high sensitivity C-reactive protein, *LDL-C* low-density lipoprotein cholesterol, *MetX* metabolic syndrome X, *Statin* HMG-CoA reductase inhibitors

^a^p value for Kruskal–Wallis test among the four groups

^b^p value Mann–Whitney U test for CSX (+) and MetX (+) vs. CSX (−) and MetX (−)

Mann–Whitney U test, Bonferroni corrected p cut-off value for significance <0.0083

### Circulating adipokine levels and insulin-resistance index in subjects with and without cardiac or metabolic syndrome X

Circulating adiponectin and leptin were significantly different among the four groups (p < 0.001) (Table 2). Subjects with combined MetX and CSX had a lower circulating adiponectin level (median 2.8 vs. 12.3 μg/mL, p < 0.001) than those without MetX and CSX (Table 2). In pairwise comparisons, subjects with CSX alone (median 3.4 μg/mL, p < 0.001) or MetX alone (median 4.0 μg/mL, p = 0.002) also had a significantly lower adiponectin level than the control group (median 12.3 μg/mL) (Table 2). In terms of circulating leptin, subjects with combined MetX and CSX (median 8.7 ng/mL, p < 0.001) or MetX alone (median 8.1 ng/mL, p = 0.002) had a significantly higher leptin than the control group (median 3.3 ng/mL) (Table 2). The HOMA-IR was significantly different among the four groups (p = 0.009), and subjects with combined MetX and CSX had a significantly higher HOMA-IR than the control (p = 0.005) (Table 2). Moreover, combined MetX and CSX subjects had a similar circulating adiponectin, leptin and IR index comparing with those who had only either one syndrome X (Table 2).

**Table 2:** Circulating adipokine levels and insulin-resistance index in subjects with and without cardiac or metabolic syndrome X

	CSX (−) MetX (−) (N = 39)	CSX (+) MetX (−) (N = 35)	CSX (−) MetX (+) (N = 15)	CSX (+) MetX (+) (N = 24)	*p* ^a^	*p* ^b^
Adiponectin (μg/mL)	12.3 (7.0, 18.9)	3.4 (2.3, 7.2)	4.0 (2.0, 9.1)	2.8 (1.7, 7.7)	<0.001	<0.001
Leptin (ng/mL)	3.3 (1.3, 8.1)	5.1 (3.1, 11.0)	8.1 (5.1, 18.5)	8.7 (6.3, 17.4)	<0.001	<0.001
HOMA-IR	1.04 (0.72, 2.47)	1.93 (1.28, 2.62)	2.40 (1.78, 4.21)	2.23 (1.48, 5.89)	0.009	0.005

Data are expressed as median (interquartile range, 25th percentile to 75th percentile) because of a non-normal distribution

*CSX* cardiac syndrome X, *MetX* metabolic syndrome X, *HOMA-IR* homeostasis model assessment (HOMA) index of insulin resistance = (fasting glucose mg/dL × fasting insulin μU/mL)/405

Mann–Whitney U test for comparisons below (Bonferroni corrected p cut-off value for significance <0.0083)

Adiponectin

Adiponectin CSX(−) MetX (−) vs. CSX (+) MetX (+), p < 0.001

Adiponectin CSX(−) MetX (−) vs. CSX (+) MetX (−), p < 0.001

Adiponectin CSX (−) MetX (−) vs. CSX (−) MetX (+), p = 0.002

Adiponectin CSX (+) MetX (−) vs. CSX (+) MetX (+), p = 0.260

Adiponectin CSX (−) MetX (+) vs. CSX (+) MetX (+), p = 0.326

Adiponectin CSX (−) MetX (+) vs. CSX (+) MetX (−), p = 0.924

Leptin

Leptin CSX(−) MetX (−) vs. CSX (+) MetX (+), p < 0.001

Leptin CSX(−) MetX (−) vs. CSX (+) MetX (−), p = 0.067

Leptin CSX (−) MetX (−) vs. CSX (−) MetX (+), p = 0.002

Leptin CSX (+) MetX (−) vs. CSX (+) MetX (+), p = 0.030

Leptin CSX (−) MetX (+) vs. CSX (+) MetX (+), p = 0.603

Leptin CSX (−) MetX (+) vs. CSX (+) MetX (−), p = 0.105

HOMA-IR

HOMA-IR CSX(−) MetX (−) vs. CSX (+) MetX (+), p = 0.005

HOMA-IR CSX(−) MetX (−) vs. CSX (+) MetX (−), p = 0.023

HOMA-IR CSX (−) MetX (−) vs. CSX (−) MetX (+), p = 0.044

HOMA-IR CSX (+) MetX (−) vs. CSX (+) MetX (+), p = 0.151

HOMA-IR CSX (−) MetX (+) vs. CSX (+) MetX (+), p = 0.862

HOMA-IR CSX (−) MetX (+) vs. CSX (+) MetX (−), p = 0.130

^a^p value for Kruskal–Wallis test among the four groups

^b^p value Mann–Whitney U test for CSX (+) and MetX (+) vs. CSX (−) and MetX (−)

### Binary logistic regression analysis of variables associated with the diagnosis of “combined cardiac and metabolic syndrome X (double syndrome X)”

In the analysis, which included five components of MetX, cigarette smoking status, IR index, circulating adipokines, age and gender, hypertension (odds ratio 14.453, p = 0.003) and triglycerides (odds ratio 1.011, p = 0.024) were positively associated with the presence of combined CSX and MetX (Table 3).

**Table 3:** Binary logistic regression analysis of independent variables associated with “combined cardiac and metabolic syndrome X”

Factors	p value	OR	95 % CI	
			Lower limit	Upper limit
Hypertension (with vs. without)	0.003	14.453	2.491	83.868
Body mass index (kg/m^2^)	0.593	1.055	0.866	1.286
Fasting glucose (mg/dL)	0.291	1.014	0.989	1.039
HDL-C (mg/dL)	0.200	0.949	0.876	1.028
Triglycerides (mg/dL)	0.024	1.011	1.001	1.021
Current smoking (yes vs. no)	0.069	4.969	0.883	27.974
HOMA-IR	0.881	0.981	0.763	1.261
Leptin (ng/mL)	0.758	1.007	0.965	1.050
Adiponectin (μg/mL)	0.638	0.978	0.893	1.072
Age (years)	0.491	1.025	0.955	1.100
Gender (male vs. female)	0.226	0.320	0.051	2.021

Dependent variable: “combined cardiac and metabolic syndrome X”

Independent variables: components of metabolic syndrome, adipokines, insulin-resistance index, smoking status, age, gender

*CI* confidence interval, *HDL-C* high-density lipoprotein cholesterol, *HOMA-IR* homeostasis model assessment (HOMA) index of insulin-resistance = (fasting glucose mg/dL × fasting insulin μU/mL)/405, *OR* odds ratio

### Binary logistic regression analysis of variables associated with the diagnosis of “CSX without MetX”

Using “CSX without MetX” as the dependent variable, we found that higher serum triglycerides (odds ratio 0.979, p = 0.001) and higher circulating adiponectin (odds ratio 0.876, p = 0.002) were negatively associated with the diagnosis of “CSX without MetX” (Table 4).

**Table 4:** Binary logistic regression analysis of independent variables associated with “cardiac syndrome X without metabolic syndrome X”

Factors	p value	OR	95 % CI	
			Lower limit	Upper limit
Hypertension (with vs. without)	0.726	0.821	0.273	2.470
Body mass index (kg/m^2^)	0.443	0.941	0.807	1.098
Fasting glucose (mg/dL)	0.931	0.999	0.981	1.018
HDL-C (mg/dL)	0.284	1.026	0.979	1.076
Triglycerides (mg/dL)	0.001	0.979	0.967	0.991
Current smoking (yes vs. no)	0.842	0.862	0.201	3.699
HOMA-IR	0.349	0.868	0.645	1.168
Leptin (ng/mL)	0.137	1.033	0.990	1.079
Adiponectin (μg/mL)	0.002	0.876	0.807	0.951
Age (years)	0.384	1.023	0.972	1.077
Gender (male vs. female)	0.261	2.108	0.575	7.731

Dependent variable: “cardiac syndrome X without metabolic syndrome X”

Independent variables: components of metabolic syndrome, adipokines, insulin-resistance index, smoking status, age, gender

*CI* confidence interval, *HDL-C* high-density lipoprotein cholesterol, *HOMA-IR* homeostasis model assessment (HOMA) index of insulin-resistance = (fasting glucose mg/dL × fasting insulin μU/mL)/405, *OR* odds ratio

### Binary logistic regression analysis of variables associated with the diagnosis of “MetX without CSX”

Using “MetX without CSX” as the dependent variable, we found that BMI was positively correlated (odds ratio 1.529, p = 0.006), while higher HDL-C (odds ratio 0.915, p = 0.049) and male gender (odds ratio 0.118, p = 0.037) were negatively associated with the diagnosis of “MetX without CSX” (Table 5).

**Table 5:** Binary logistic regression analysis of independent variables associated with “metabolic syndrome X without cardiac syndrome X”

Factors	p value	OR	95 % CI	
			Lower limit	Upper limit
Hypertension (with vs. without)	0.702	0.749	0.171	3.285
Body mass index (kg/m^2^)	0.006	1.529	1.131	2.066
Fasting glucose (mg/dL)	0.240	1.014	0.991	1.037
HDL-C (mg/dL)	0.049	0.915	0.838	1.000
Triglycerides (mg/dL)	0.116	1.008	0.998	1.019
Current smoking (yes vs. no)	0.605	0.580	0.073	4.578
HOMA-IR	0.304	0.866	0.658	1.139
Leptin (ng/mL)	0.089	0.928	0.852	1.011
Adiponectin (μg/mL)	0.743	0.984	0.896	1.081
Age (years)	0.879	0.994	0.918	1.076
Gender (male vs. female)	0.037	0.118	0.016	0.881

Dependent variable: “metabolic syndrome X without cardiac syndrome X”

Independent variables: components of metabolic syndrome, adipokines, insulin-resistance index, smoking status, age, gender

*CI* confidence interval, *HDL-C* high-density lipoprotein cholesterol; HOMA-IR: homeostasis model assessment (HOMA) index of insulin-resistance = (fasting glucose mg/dL × fasting insulin μU/mL)/405, *OR* odds ratio

## Discussion

The main finding of our study was that subjects with combined MetX and CSX had a significantly higher HOMA-IR, a higher circulating leptin, but a lower circulating adiponectin level than those without MetX and CSX. Combined syndrome X did not confer more changes on adipokines or IR index comparing with those with only one syndrome X. Serum triglycerides and hypertension were independently associated with the diagnosis of combined MetX and CSX in subjects who underwent CAG but without stenosis.

Only a few small-scale studies have investigated common etiologies in CSX and MetX, which bore similar names of syndrome X. The most common observation was the presence of IR in both of these syndromes [14, 16]. Our study found that insulin-resistance index was significantly higher in subjects with combined double syndrome X than the control group (Table 2), which further corroborated that IR contributed to the pathogenesis of both CSX and MetX. However, combined syndrome X did not result in more changes on IR index comparing with those with only one syndrome X (Table 2).

There have been very few reports about the circulating adipokines in combined MetX and CSX subjects. Our group previously reported that subjects with CSX had a significantly lower serum adiponectin than those of the control group [22]. Subjects with MetX also had decreased circulating adiponectin [17, 19]. The present study extended the investigation into combined MetX and CSX and revealed a similar lower expression of adiponectin in combined syndrome X as well as in MetX or CSX alone groups (Table 2). Previous study by Eroglu et al. [29] showed adiponectin levels were lower in female patients with normal coronary angiograms but impaired coronary flow reserve than those with normal reserve. In addition, adiponectin levels correlated with coronary flow reserve [29]. Their findings along with our observations [22] indicated the potential role of adiponectin in mediating coronary flow and microvascular dysfunction in CSX subjects. Leptin expression was higher in subjects with CSX as reported by our research group [22] and others [15]. A number of studies have revealed that increased plasma leptin concentration is correlated with higher blood pressure [30], higher cholesterol level, and morbid obesity [19]. High levels of leptin are believed to be associated with lower arterial distensibility, an index of circulatory function relevant to the atherosclerotic process [31]. As leptin was correlated with several conventional atherosclerotic risk factors as well as arterial distensibility, it is reasonable that subjects with combined CSX and MetX had a higher leptin expression (Table 2). Our study also disclosed that combined syndrome X did not contribute to more changes on adipokines comparing with those with only one syndrome X (Table 2).

A novel finding of this study is that hypertension and serum triglycerides were independent variables associated with the diagnosis of combined CSX and MetX (Table 3). Hypertension and hypertriglyceridemia per se are components of MetX. Hypertriglyceridemia was a reflection of IR and decreased adipose tissue lipoprotein lipase activity [32]. CSX subjects were also characterized by the presence of IR and related adiposity [14, 16]. Hypertension and associated left ventricular hypertrophy impaired endothelium-mediated relaxation in human coronary resistance vessels [33–35]. Our previous study disclosed that hypertension is a significant predictor for the diagnosis of CSX [22]. In this study, there were significant trends toward higher triglycerides and higher percentage of associated hypertension in the order of control, CSX, MetX and combined double syndrome X (Table 1). In contrast, IR index and circulating adipokines were similar among subjects with CSX, MetX or combined double syndrome X (Table 2). Triglycerides levels and hypertension showed trends toward higher value and association in those with combined double syndrome X (Tables 1, 3). It may imply that decreased lipoprotein lipase activity, endothelium dysfunction or sympathetic over-activity contribute to combined double syndrome X [32, 35, 36]. However, the finding demands further in vitro and in vivo mechanism investigation.

In addition, our study also disclosed that higher serum triglycerides and higher circulating adiponectin were negatively associated with the diagnosis of “CSX without MetX” (Table 4). It would implicate that CSX is closely associated with low adiponectin related endothelium dysfunction and MetX is strongly related with decreased lipoprotein lipase activity related high triglycerides [1, 22, 29]. Moreover, we found that higher BMI was positively correlated while higher HDL-C and male gender were negatively associated with the diagnosis of “MetX without CSX” (Table 5). This would imply BMI and HDL-C are useful differential factors between MetX and CSX.

There are several limitations of this study. This was a retrospective analysis from a specific time-frame of the cardiac catheterization data bank in a single medical center, thus the recruited patient number and study power could not be specified beforehand. According to the data of circulating adiponectin of CSX, MetX, and the controls from the publications [19, 22], we think this retrospective analysis could have a study power around 0.75–0.8. Moreover, this was an observational analysis study without further investigation of the disease mechanism.

In conclusion, subjects with combined MetX and CSX had a significantly higher HOMA-IR, a higher circulating leptin, but a lower circulating adiponectin level than those without MetX and CSX. Combined MetX and CSX did not confer more changes on adipokines or IR index comparing with those with only one syndrome X. Serum triglycerides and hypertension were independently associated with the diagnosis of combined MetX and CSX among subjects who underwent CAG but without stenosis.
